# Identification of potential diagnostic and prognostic biomarkers for papillary thyroid microcarcinoma (PTMC) based on TMT-labeled LC–MS/MS and machine learning

**DOI:** 10.1007/s40618-022-01960-x

**Published:** 2022-11-23

**Authors:** J. Li, L. Mi, B. Ran, C. Sui, L. Zhou, F. Li, G. Dionigi, H. Sun, N. Liang

**Affiliations:** 1 Division of Thyroid Surgery, Jilin Provincial Key Laboratory of Surgical Translational Medicine, Jilin Provincial Precision Medicine Laboratory of Molecular Biology and Translational Medicine on Differentiated Thyroid Carcinoma, China-Japan Union Hospital of Jilin University, 126 Xiantai Street, Changchun, 130033 Jilin China; 2 Division of General and Endocrine Surgery, Department of Medical Biotechnology and Translational Medicine, Istituto Auxologico Italiano IRCCS, University of Milan, Milan, Italy

**Keywords:** Thyroid cancer, Lymph node metastasis, Proteomics, PRM, Machine learning, Nomogram, TCGA database

## Abstract

**Objectives:**

To explore the molecular mechanisms underlying aggressive progression of papillary thyroid microcarcinoma and identify potential biomarkers.

**Methods:**

Samples were collected and sequenced using tandem mass tag-labeled liquid chromatography–tandem mass spectrometry. Differentially expressed proteins (DEPs) were identified and further analyzed using Mfuzz and protein–protein interaction analysis (PPI). Parallel reaction monitoring (PRM) and immunohistochemistry (IHC) were performed to validate the DEPs.

**Results:**

Five thousand, two hundred and three DEPs were identified and quantified from the tumor/normal comparison group or the N1/N0 comparison group. Mfuzz analysis showed that clusters of DEPs were enriched according to progressive status, followed by normal tissue, tumors without lymphatic metastases, and tumors with lymphatic metastases. Analysis of PPI revealed that DEPs interacted with and were enriched in the following metabolic pathways: apoptosis, tricarboxylic acid cycle, PI3K-Akt pathway, cholesterol metabolism, pyruvate metabolism, and thyroid hormone synthesis. In addition, 18 of the 20 target proteins were successfully validated with PRM and IHC in another 20 paired validation samples. Based on machine learning, the five proteins that showed the best performance in discriminating between tumor and normal nodules were PDLIM4, ANXA1, PKM, NPC2, and LMNA. FN1 performed well in discriminating between patients with lymph node metastases (N1) and N0 with an AUC of 0.690. Finally, five validated DEPs showed a potential prognostic role after examining The Cancer Genome Atlas database: FN1, IDH2, VDAC1, FABP4, and TG. Accordingly, a nomogram was constructed whose concordance index was 0.685 (confidence interval: 0.645–0.726).

**Conclusions:**

PDLIM4, ANXA1, PKM, NPC2, LMNA, and FN1 are potential diagnostic biomarkers. The five-protein nomogram could be a prognostic biomarker.

**Supplementary Information:**

The online version contains supplementary material available at 10.1007/s40618-022-01960-x.

## Introduction

Papillary thyroid carcinoma (PTC) is the most common pathologic form of thyroid cancer and its incidence has increased worldwide over the past 50 years [1, 2]. Although the 10-year survival rate of PTC > is 90% [3, 4], metastasis and recurrence remain a challenge for clinicians and a burden for patients [4, 5]. Papillary thyroid microcarcinoma (PTMC) refers to PTC with a maximum diameter of less than 1.0 cm [6], and the treatment strategy is currently controversial and consists only of dynamic follow-up or active diagnosis and surgery. It is worthwhile to explore the molecular mechanisms underlying the aggressive progression and prognosis of PTMC and propose potential biomarker models [7].

With the rapid development of high-throughput proteomic techniques, proteomics has become an important strategy for determining tumor biological mechanisms and exploring potential cancer biomarkers [7]. In contrast to genomics and transcriptomics, proteomics has focused on the identification of differentially expressed proteins (DEPs) and other molecular mechanisms at both the protein and post-translational modification levels [8]. In this study, we first established the proteomic profile of PTMC, validated several target proteins with parallel reaction monitoring (PRM), and finally generated a multiprotein prognostic signature using machine learning and nomograms.

## Materials and methods

### Samples collection

All specimens were obtained from the Department of Thyroid Surgery, China-Japan Union Hospital, Jilin College. Patients with PTMC who had undergone surgery and had a pathological diagnosis were included in the study. Both tumor samples and adjacent normal samples were collected 30 min after surgery, immediately transferred to sterilized vials, frozen in liquid nitrogen, and stored at – 80 ℃.

### Ethical standards

The study was conducted in accordance with the Declaration of Helsinki (revised 2013) and approved by the China-Japan Union Hospital Institutional Review Board (No. 20220804014). Informed consent was obtained from all the patients.

### Protein extraction and TMT labeling

Tandem mass labeling (TMT) was performed for mass spectroscopy (MS) using three consecutive TMT-10-plex (catalog no. 90111, Thermo, USA) isobaric labeling kits according to the manufacturer’s protocol. For digestion, the protein solution was reduced with 5 mM dithiothreitol for 30 min at 56 °C and alkylated with 11 mM iodoacetamide for 15 min at room temperature in the dark. The protein sample was diluted to a urea concentration of less than 2 M by adding 100 mM triethylammonium bicarbonate. Finally, trypsin was added at a ratio of 1:50 trypsin to protein mass for the first overnight digest and at a ratio of 1:100 trypsin to protein mass for a second 4 h digest.

### LC–MS/MS analysis

For data acquisition, tryptic peptides were dissolved in 0.1% formic acid (solvent A) and loaded directly onto a home-built analytical reversed-phase column. The gradient included an increase from 6 to 23% solvent B (0.1% formic acid in 98% acetonitrile) over 26 min, 23–35% over 8 min, and an increase to 80% over 3 min to then remain at 80% for the final 3 min, all at a constant flow rate of 400 nL/min on a EASY-nLC 1000 UPLC system (Thermo, USA). The resulting liquid chromatography–tandem mass spectrometry data (LC–MS/MS) were processed using the MaxQuant search engine (v.1.5.2.8). Tandem mass spectra were aligned with the human Swiss-Prot database, which was concatenated with a reverse Decoy database. Trypsin/P was indicated as a cleavage enzyme, with up to four missing cleavages. The mass tolerance for precursor ions was set at 20 ppm for the first search and 5 ppm for the main search, and the mass tolerance for fragment ions was set at 0.02 Da. Carbamidomethyl on Cys was reported as a fixed modification, and 18 modifications and oxidation on Met were reported as variable modifications. The false discovery rate was set at < 1%, and the minimum score for modified peptides was set at > 40.

### Bioinformatic analysis

#### Dynamic expression model analysis (Mfuzz)

Fragments per kilobase of transcripts per million mapped reads (FPKM) from DEPs identified by the likelihood ratio test with an adjusted *P* value of < 0.05 were used for c-means clustering with the R package Mfuzz to characterize dynamic changes in expression patterns. Fuzzy c-means clustering is a soft clustering method that uses the Mfuzz algorithm with two key parameters. The algorithm iteratively assigns the profile to the cluster with the shortest Euclidean distance while minimizing an arbitrary objective function.

#### KEGG pathway annotation

The Kyoto Encyclopedia of Genes and Genomes (KEGG) database was used for protein pathway annotation. First, the KEGG online service tool KEGG Automatic Annotation Server was used to annotate the description of the KEGG database for proteins. The annotation results were mapped to the KEGG pathway database using the KEGG online service tool KEGG mapper. The circles in the figure show the enrichment of differentially expressed proteins. Red represents highly significant enrichment of differentially expressed proteins (*P* < 0.05) and blue represents nonsignificant enrichment. The size of the circle represents the fold enrichment.

#### PPI (protein–protein interaction) network analysis

The database protein numbers or sequences of the differentially expressed proteins from the different comparison groups were compared with the database of the STRING (v.10.5) protein network, and the differential protein interaction relationship was extracted using a confidence value > 0.4.

### PRM validation

PRM verification included protein extraction, trypsin digestion, LC–MS/MS analysis, and data processing using the Skyline (v.3.6) software. A validation set of 20 pairs of specimens was detected using PRM.

### Machine learning-random forest experiment

The target proteins in the two comparison groups were sorted by their characteristics and then selected and applied to the receiver operating characteristics (ROC) analysis. In general, the closer the ROC curve is to the upper left corner, the better the predictive ability of the model. An intuitive indicator is the area under the ROC curve, namely the AUC (area under the curve), where the AUC value ranges from 0.5 to 1, with 0.5 denoting a poor classifier and 1 denoting an excellent classifier. SIMCA (version 14.0) software was used for partial least squares discrimination analysis (PLS-DA). The protein of predictive variable importance in projection larger than 1 (VIP pred > 1) was considered to be meaningful for sample discrimination. Further leave-one-out (LOO) cross-validation was applied to select candidate biomarkers. The samples were trained and evaluated in a leave-one-out manner using scikit-learn python package. Logistic regression was chosen as the classifier with {\rm solver = 'liblinear’} and {\rm class weight = 'balanced’}.

### Prognostic validity of the model

The mRNA sequencing (RNA-Seq) data and corresponding clinical information of 507 patients with PTC were downloaded from The Cancer Genome Atlas (TCGA) database (https://portal.gdc.cancer.gov/repository). The dataset includes 510 PTC tissue samples and 58 adjacent normal tissue samples. Cox regression and the Kaplan–Meier method were applied to analyze the association between target proteins, clinicopathologic features, and prognosis. The RMS package (version 5.1–4; https://cran.r-project.org/web/packages/rms/index.html) was used to generate the nomogram. The calibration curves were evaluated graphically by mapping the probabilities predicted by the nomogram to the observed occurrences. A concordance index (C-index) was used to determine the discriminant ability of the nomogram.

## Results

### The proteomic profile of PTMC

To elucidate the proteomic profile of PTMC, six paired normal tumor tissues were collected and further sequenced using TMT-labeled LC–MS /MS. Depending on the status of lymphatic metastases, six paired samples were divided into N0 and N1 groups. The N1 group included patients with lymphatic metastases, while the N0 group included patients without lymphatic metastases. In this study, we paid more attention to the following two differences: one was the difference between tumor and normal (tumor/normal group), and the other was the discrepancy between the N1 group and the N0 group (N1/N0 comparison group).

**Fig. 1:**
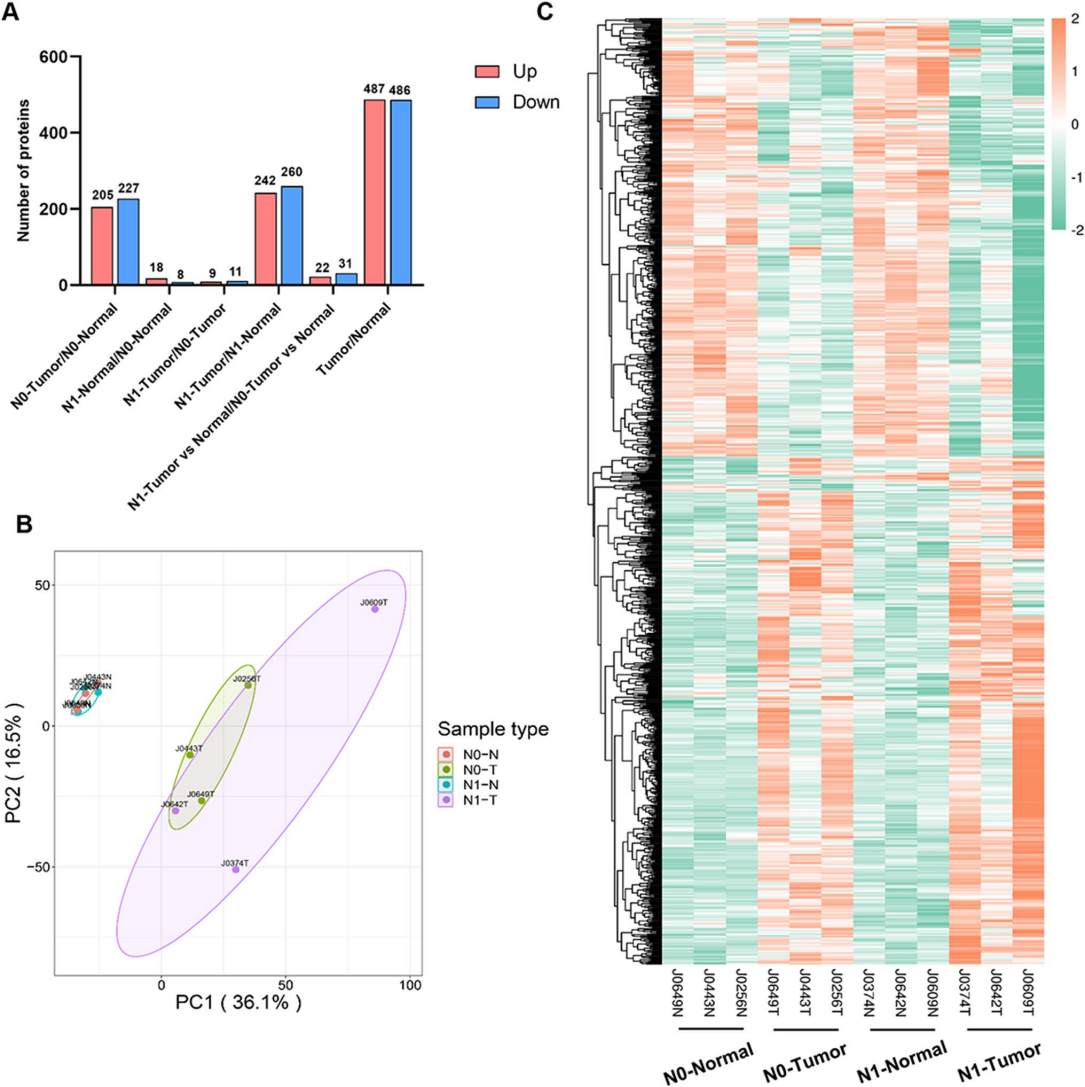
Schematic representation of the overall study design and differentially expressed proteins between Tumor and Normal tissues using liquid chromatography–tandem mass spectrometry (LC–MS/MS) analysis. **A** The bar chart shows the number of differentially expressed proteins (DEPs) in each functional group. **B** Principal component analysis (PCA) plot showing unsupervised clustering among the 12 samples that combined Tumor and Normal tissues. **C** Heat map representation of total expressed proteins in all comparison groups. Significantly enriched downregulated (green) and upregulated (red) proteins. The dendrograms represent the classification of proteins

In this study, a total of 5203 proteins were identified and quantified with a fold change threshold of > 1.30 or < 0.67. In the tumor/normal group, 487 proteins were upregulated and 486 proteins were significantly downregulated compared with the normal tissues. Similarly, more than 200 proteins were upregulated and downregulated in the N0 tumor/N0 normal group and the N1 tumor/N1 normal group, respectively. However, contrary to our expectations, only 20 DEPs were observed in the N1 tumor/N0 tumor group, and 53 DEPs were detected in the standardized N1 tumor vs normal/N0 tumor vs normal group (Fig. 1A). To further investigate the possible DEPs between tissues with lymph node metastasis and negative tissues, we considered the following three comparisons: the standardized N1 tumor vs. normal/N0 tumor vs. normal group, the N1 tumor/N0 tumor group, and DEPs between N1 tumor/N1 normal and N0 tumor/N0 normal. Principal component analysis showed a clear distinction between tumor and normal samples, but an overlap between N1 tumor and N0 tumor (Fig. 1B). As shown in Fig. 1C, hierarchical cluster analysis revealed a clear differential expression pattern between tumor and normal samples. However, when comparing N0 tumor and N1 tumor, the difference was not as clear as when comparing tumor and normal, but a slight change was also observed.

### The dynamic expression patterns and associated pathways in the normal-tumor-metastasis progression of PTMC

**Fig. 2:**
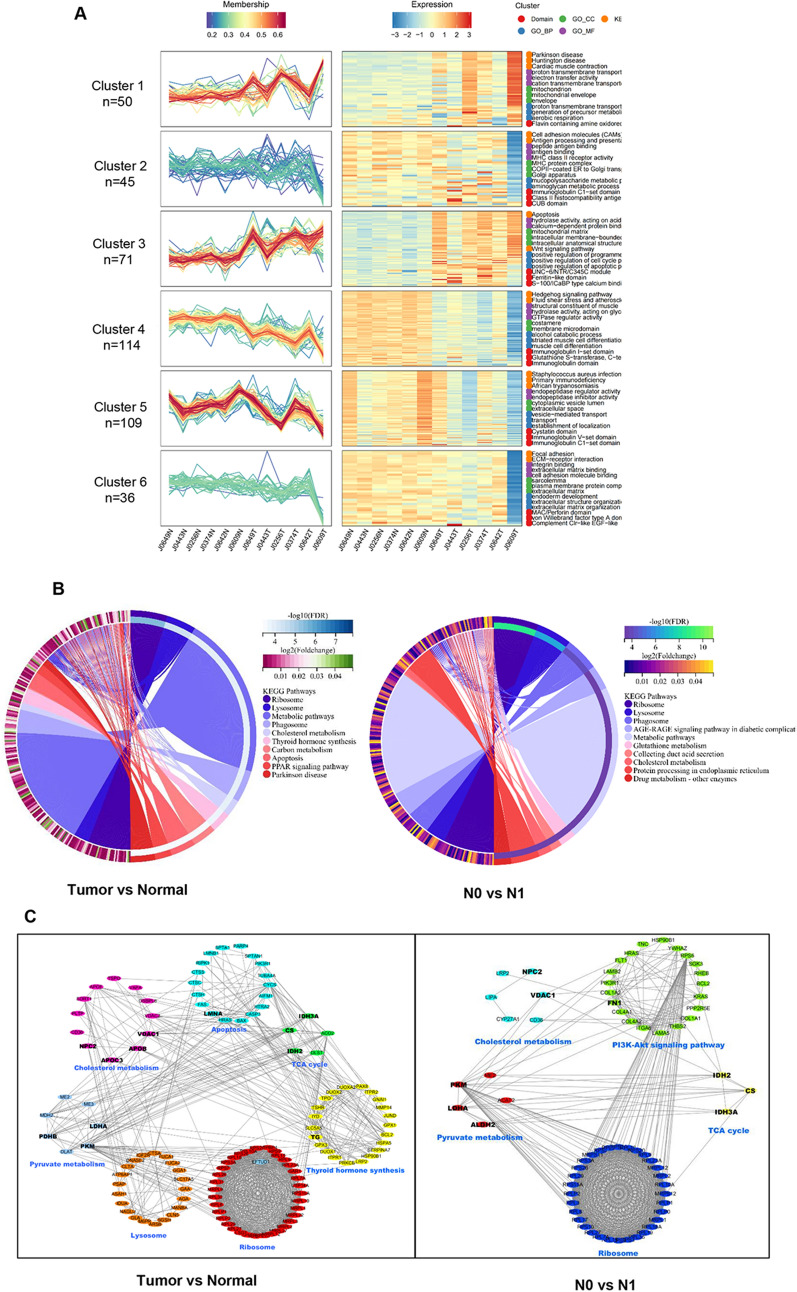
Results of the clustering analysis using Mfuzz and pathways enrichment analyses highlighting the significance of differentially expressed proteins (DEPs) using the Kyoto Encyclopedia of Genes and Genomes (KEGG) and protein–protein interaction (PPI). **A** Mfuzz clustering analysis of DEPs. Six clusters are identified using Mfuzz software. Proteins in clusters 1 and 3 show a consistently increasing trend from Normal tissues to N0-Tumor tissues which represent the appearance of papillary thyroid microcarcinoma (PTMC). Proteins in clusters 4 and 5 show a consistently decreasing trend from N0-Tumor to N1-Tumor tissues, which represent the progression of PTMC. **B** The KEGG pathway enrichment analysis of DEPs. The left pie chart shows the top 10 distribution of pathways in each KEGG category of the Tumor/Normal comparison group. The right panel shows the top 10 KEGG pathways in the N1/N0 comparison group. **C** PPI network construction. The left PPI network is based on the proteins enriched in apoptosis, thyroid hormone synthesis, cholesterol metabolism, lysosome, pyruvate metabolism, and ribosome, which are selected from the top 10 pathways in the Tumor/Normal KEGG pathway and important pathways in previous research. The right PPI network is based on the proteins enriched in ribosome, pyruvate metabolism, cholesterol metabolism, tricarboxylic acid (TCA) cycle, and PI3K-Akt signaling pathway, which are selected from the top 10 pathways in the N0/N1 comparison group and important pathways in previous research. The proteins marked with a bigger size are selected for parallel reaction monitoring (PRM) and immunohistochemical (IHC) protein analyses

To analyze the dynamic expression patterns of DEPs during the progression of PTMC, the 5203 DEPs were divided into 6 patterns (clusters 1 to 6) using Mfuzz analysis. As shown in Fig. 2A, DEP clusters were enriched according to progression status, followed by normal tissue (N0 normal and N1 normal), tumors without lymphatic metastases (N0 tumor), and tumors with lymphatic metastases (N1 tumor). The expression patterns of clusters 1 and 3 gradually increased with disease progression. These DEPs were significantly enriched in Parkinson’s disease, Huntington’s disease, myocardial contraction, apoptosis, and Wnt signaling. Meanwhile, clusters 4 and 5 were initially downregulated during the transition from normal to tumor, but were upregulated again during the transition from N0 tumor to N1 tumor. These related DEPs were mainly enriched in Hedgehog signaling, fluid shear stress, atherosclerosis, African trypanosomiasis, and complement and coagulation. This suggests that these DEPs, as well as their associated signaling pathways, may play an important role in the progression of PTMC.

To investigate the specific functions of DEPs, KEGG pathway analysis was performed. As shown by the circle map in Fig. 2B, DEPs in the tumor/normal group were mainly enriched in ribosome, lysosome, phagosome, cholesterol metabolism, and thyroid hormone synthesis. Similarly, DEPs from the N1 vs. N0 comparison group were mainly enriched in AGE-RAGE signaling pathway, cholesterol metabolism, pyruvic acid pathway, etc. In addition, PPI was used to investigate the possible network interactions of the DEPs (Fig. 2C). The DEPs from the tumor/normal group interacted and were enriched in the following metabolic pathways: apoptosis, lysosome, ribosome, cholesterol metabolism, pyruvate metabolism, and thyroid hormone synthesis. Similarly, DEPs from the N1/N0 comparison group interacted and were enriched in the tricarboxylic acid (TCA) cycle and PI3K-Akt pathway. Based on the above analysis and literature review, 20 candidate proteins were selected as targets for validation.

### Validation of targeted proteins

**Fig. 3:**
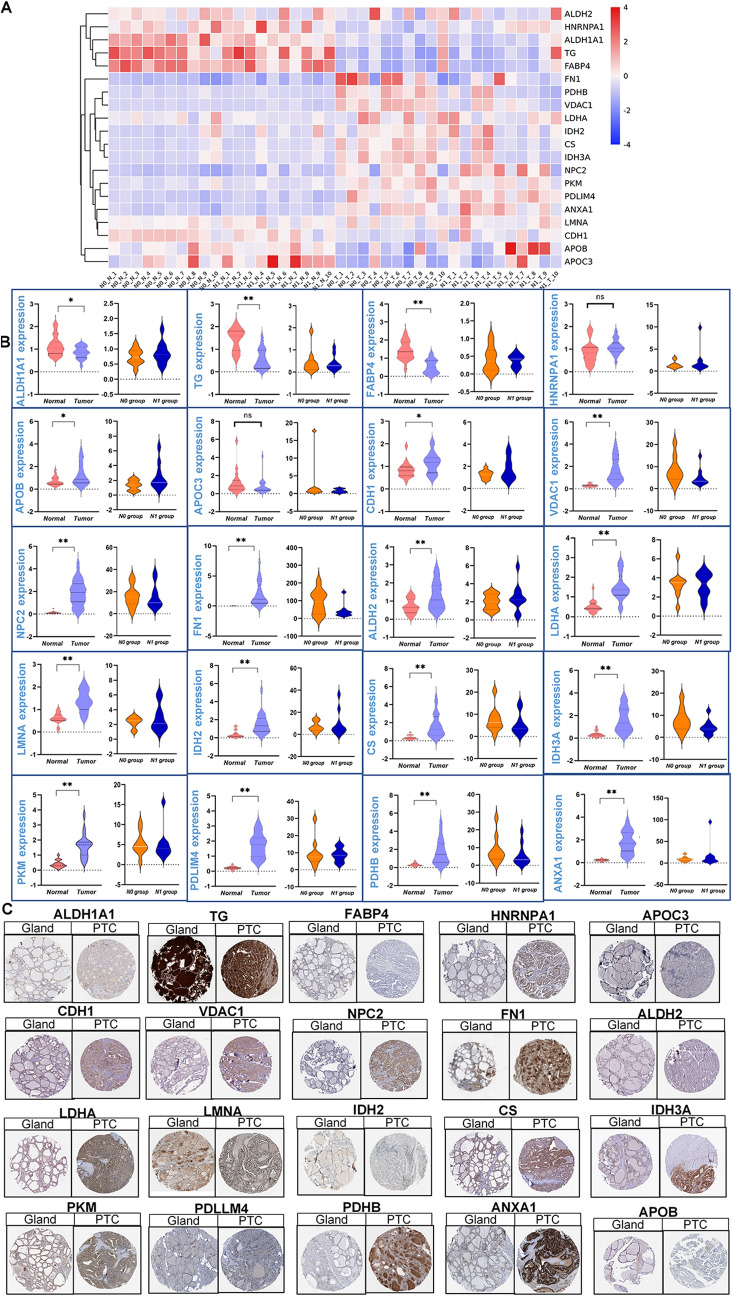
Confirmation of proteomic alterations using parallel reaction monitoring (PRM) and immunohistochemical (IHC) protein analyses. **A** Heatmap representation of the overall abundance profile of all 20 detected proteins in the 20 PRM pairs of papillary thyroid microcarcinoma (PTMC) tissues and adjacent noncancerous tissues. Red represents upregulation, and blue represents downregulation. **B** Violin plots represent the significantly different expression levels of PRM proteins between the Tumor/Normal comparison group and the N0/N1 comparison group. One asterisk indicates *P* < 0.05–0.01; two asterisks indicate *P* < 0.01–0.001. **C** IHC expression of PRM protein between PTC tissues and gland tissues

To validate the targeted proteins, 20 paired samples were collected from PTMC and further analyzed with PRM. The basic clinical information of the patients is shown in suppletment Table 1. Similarly to the original PTMC sequencing, half of the patients had lymph node metastases (N1 stage) in the validation, but only two out of ten were in the progressive stage (N1b stage). Indeed, the heat map of PRM showed an obvious trend and pattern that changed according to the progression normal-tumor-metastasis (Fig. 3A). The detailed statistical results showed that 18 of the 20 target proteins were consistent with the previous sequencing data (Fig. 3B). Unfortunately, the differential expression of DEPs between N1- and N0-stage patients was not obvious, which may be due to the lower proportion of N1b-stage patients. In addition, immunohistochemistry (IHC) was performed to investigate the different expression levels. As shown in Fig. 3C, most results were consistent with PRM. Take NPC2 and PHDB as examples, those two genes were higher expressed in PTC tissues, compared with the thyroid gland. The association between target proteins and clinical features needs to be further validated using a larger number of samples.

### Predictive performance of biomarkers calculated via machine learning

**Fig. 4:**
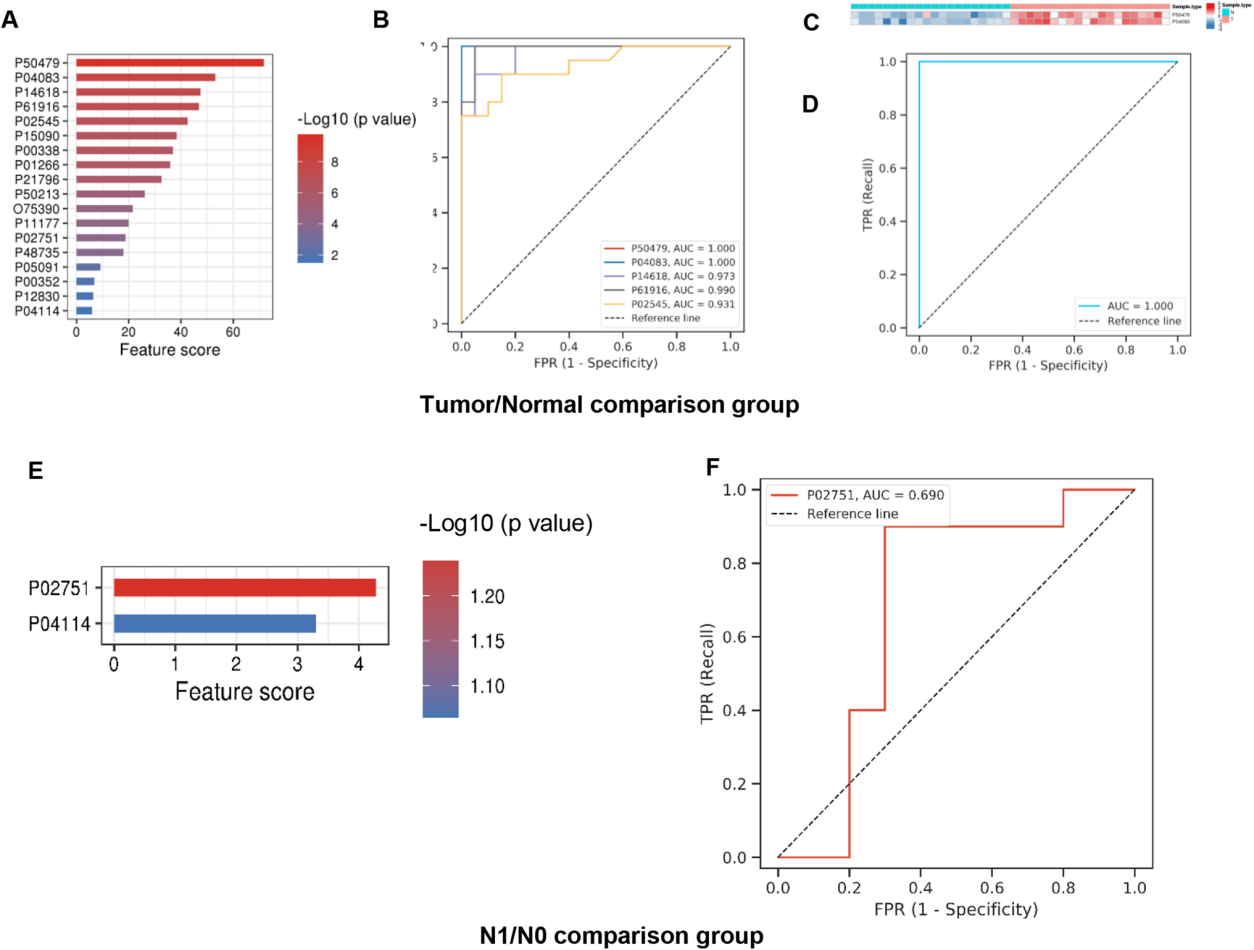
Machine learning model to identify potential target proteins of onset and progression in papillary thyroid microcarcinoma (PTMC). **A** Overall distribution of feature scores among proteins in normal and tumor tissues in the training cohort. **B** The best performance of the top five proteins for predicting normal and tumor tissues of PTMC. **C** Machine learning results predicting tumor from normal tissues of PTMC. **D** Overall distribution of feature scores among proteins in N0/N1 comparison group in the training cohort. **E** The performance of FN1 for predicting metastasis of PTMC

Furthermore, using machine learning strategies based on the PRM results, we evaluated the predictive power of the validated proteins (Fig. 4). In distinguishing between tumor and normal nodules, the following five proteins showed the best performance: P50479 (PDLIM4), P04083 (ANXA1), P14618 (PKM), P61916 (NPC2), and P02545 (LMNA). All AUCs of these five proteins were greater than 0.9 (Fig. 4A and B). In addition, we further established a possible clinical predictive model by machine learning based on the PRM sequencing result. As illustrated in Fig. 4C, P50479 (PDLIM4) together with P04083 (ANXA1) could distinguish benign nodule from malignant nodule, and the AUC was as high as 1.00. Similarly, we evaluated the predictive power of the validated proteins to distinguish patients with lymph node metastases (N1) from patients without lymph node metastases (N0). Among them, P02751 (FN1) was the most relevant protein with an AUC of 0.690 (Fig. 4D and E). These results indicate its potential role as a biomarker.

### Performance of the five-protein panel to predict prognosis

**Fig. 5:**
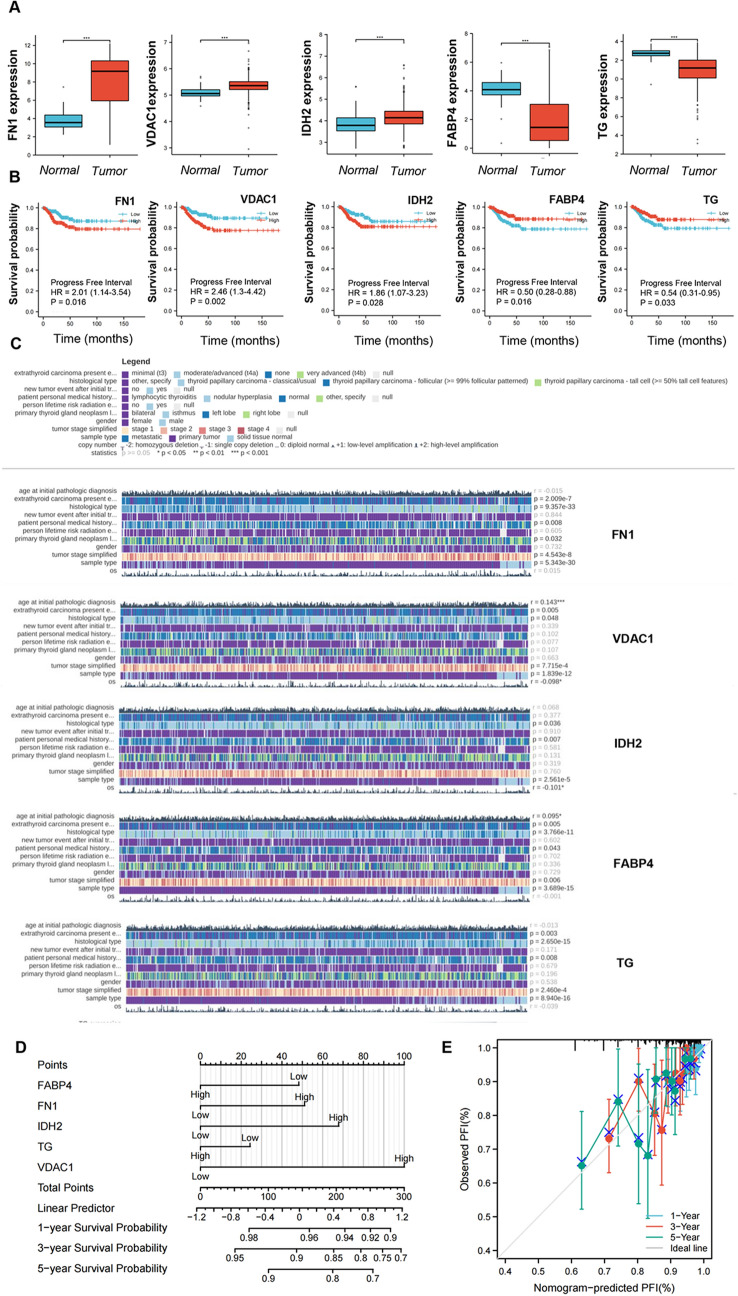
The potential target proteins showed different clinical characteristics in The Cancer Genome Atlas (TCGA) and established a nomogram for predicting the probability of 5-year progression-free interval (PFI) for patients with papillary thyroid carcinoma (PTC). **A** Expression levels of FABP4, FN1, IDH2, TG, and VDAC1 in TCGA thyroid cancer cohort with normal and tumor tissues were analyzed. **B** Survival curves of PFI between FABP4, FN1, IDH2, TG, and VDAC1-high and -low patients with PTC. **C** Relationships between FABP4, FN1, IDH2, TG, and VDAC1 and clinicopathological features. **D** A nomogram for predicting the probability of 5-year PFI for patients with PTC. **E** Calibration plots of the nomogram for predicting the probability of PFI at 5 years and the relationship between risk score and clinical information

**Fig. 6:**
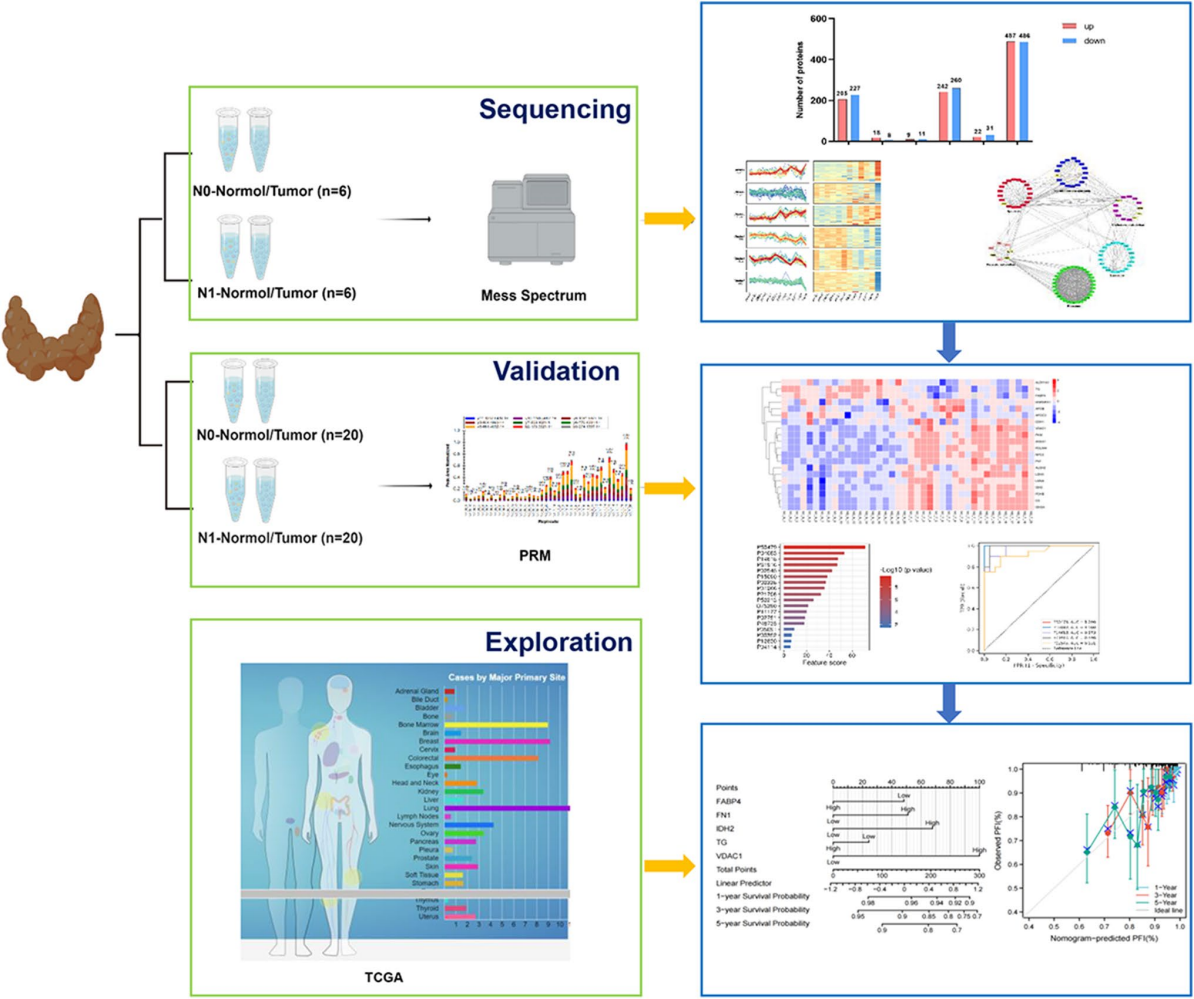
The schematic workflow of the study

To investigate their prognostic value in thyroid cancer, we further evaluated 18 validated proteins from the TCGA database. Interestingly, five proteins showed a potential prognostic role: FN1, IDH2, VDAC1, FABP4, and TG (Fig. 5). First, FN1, IDH2, and VDAC1 were more highly expressed in thyroid cancer tissues than in normal tissues; however, FABP4 and TG were less highly expressed in thyroid cancer tissues (Fig. 5A). Consequently, the higher expression of FN1, IDH2, or  VDAC1 meant a worse prognosis, as indicated by the 5-year progression-free interval (PFI). In contrast, lower expression of FABP4 and TG showed a worse prognosis (Fig. 5B). Moreover, the expression of the five proteins was associated with some clinicopathologic features, such as simplified tumor stage, extra-thyroidal carcinoma, and histologic type (Fig. 5C). To comprehensively assess the prognosis of a thyroid cancer patient, we attempted to construct a nomogram depending on the expression of these five proteins based on the above analysis (Fig. 5D). For example, a thyroid cancer patient with high FN1 risk (51 points) and high VDAC1 risk (100 points) received a total score of 151, and the 1-, 3-, and 5-year survival rates were 96%, 90%, and 85%, respectively. As shown in Fig. 5E, the predictive accuracy of this nomogram was good, as indicated by the higher C-index of 0.685 (confidence interval: 0.645–0.726). These results indicate that the five-protein nomogram has good predictive and prognostic performance. The schematic workflow of the study was shown in Fig. 6.

## Discussion

In this study, we first established the proteomic profile of PTMC, validated several target proteins using PRM and IHC analyzes, identified potential diagnostic markers using machine learning, and constructed a nomogram of five proteins to predict prognosis.

Proteomics has been used for more than 20 years. Early research was based on simple Western blot technology, and later mass spectrometry was used to identify proteins [9]. However, the limited sequencing depth and the number of identified proteins in the initial mass spectrometry technology have led to bottlenecks in proteomics research [10]. In recent years, with the rapid development of mass spectrometry technology, high-throughput protein identification has been achieved, and proteomics research has entered a new era [11]. At present, the application of high-throughput proteomics technology in cancer mainly involves revealing the mechanism of tumorigenesis and development, searching for specific biomarkers, elucidating the mechanism of drug resistance, and identifying new therapeutic targets [12]. Early detection and diagnosis of tumors is helpful for timely medical intervention and greatly improves survival and quality of life. Thyroid cancer is a common malignant endocrine tumor and its incidence is increasing annually [13, 14]. Understanding the pathogenesis of thyroid cancer is essential for improving the accuracy of diagnosis, precise risk stratification, and realizing personalized treatment. Recently, with the consistent development of mass spectrometry technologies, a variety of proteomic analysis methods based on different sample types (cells, tissues, serum, and urine) have been applied to the study of thyroid cancer, actively promoting the development of accurate diagnosis and treatment of thyroid cancer through elucidation of pathogenesis, diagnostic classification, prognosis prediction, targeted therapy, etc. [15–17]. In our study, 5203 DEPs were identified and quantified from the tumor/normal comparison group or the N1/N0 comparison group. Mfuzz analysis showed that clusters of DEPs were enriched according to progressive status, followed by normal tissue, tumors without lymphatic metastases, and tumors with lymphatic metastases. Analysis of PPI revealed that DEPs interacted and were enriched in the following signaling pathways: apoptosis, TCA cycle, PI3K-Akt pathway, cholesterol metabolism, pyruvate metabolism and thyroid hormone synthesis. The detailed molecular mechanisms and functions need to be further validated and explored both at the cellular level and in animal models.

Based on machine learning, PDLIM4, ANXA1, PKM, NPC2 and LMNA are potential diagnostic biomarkers. FN1 showed good results in distinguishing patients with lymph node metastases (N1) from N0. FN1 is a fibroadenoma protein involved in cell adhesion and migration. This may affect wound healing and the host immune system [18]. Since it is a glycoprotein involved in cell adhesion and migration, it is thought to be associated with signaling pathways related to cancer [19]. At the same time, FN1 has been shown in vitro to promote PTC progression, cell migration, and invasion as a marker of epithelial-mesenchymal transition [20]. In a previous study, FN1 was found to stimulate cell proliferation and inhibit apoptosis via the PI3K-Akt signaling pathway [21]. Some researchers found that overexpression of FN1 was an important determinant of aggressive progression of thyroid cancer [22, 23]. In contrast, the other professors showed that FN1 expression correlated with the prognosis and degree of immune infiltration in thyroid cancer, suggesting that FN1 expression can be used as an immune-related biomarker and therapeutic target in thyroid cancer [24]. Similar to Wei et al. quantitative proteomic analysis of sporadic medullary thyroid carcinomas also confirmed changes in the expression of several known biomarkers and identified FN1 as a new prognostic biomarker [25]. The results showed that FN1 could be a potential biomarker for predicting the progression of thyroid cancer, regardless of tumor classification. Therefore, FN1 may be a potential diagnostic marker. In addition, we tried to explore the possible differential expression of the potential biomarkers within the gender and age groups. The age group was divided based on the average age of 40. FN1 were higher expressed in the patients aged over 40, compared with patients aged less than 40. However, no obvious difference was detected for the other potential biomarkers within comparison. However, in this study, there was an obvious selection bias for patients’ enrollment. It’s very necessary for us to further explore whether there is a predisposition in a larger cohort.

Finally, using the TCGA database, we constructed a nomogram based on the prognosis-related validated DEPs, which showed good performance. In a previous study using the nomogram, clinicians were able to create a risk stratification system to better assess patients who would benefit from surgery for prostate cancer. In addition, nomograms are more quantitative and intuitive, making them more convenient for clinicians [26]. It has also been suggested that the nomogram accurately predicts the malignancy risk of thyroid nodules with indeterminate cytology based on clinical, cytologic, and ultrasonographic features [27]. In our study, the nomogram for 5-year PFI could help predict the severity of PTMC. Voltage-dependent anion channel 1 (VDAC1) is involved in cancer metabolism via its modulatory role in the transport of various metabolites [28]. In many cancers, the interaction of VDAC1 with hexokinase (HK) converts glucose to glucose-6-phosphate through phosphorylation, which initiates glycolysis, contributes to the unhindered growth of cancer cells, and inhibits apoptosis [29]. VDAC1 is involved in numerous biological processes, including tumor invasion, migration, metastasis, and proliferation [30]. In thyroid cancer cells, the resulting dysfunction of primary cilia leads to marked upregulation of VDAC1 genes and proteins, VDAC1 oligomerization, and apoptotic cell death. Loss of function of primary cilia in differentiated thyroid cancer cells increases VDAC1 oligomerization and induces mitochondria-dependent apoptosis [28]. Therefore, there is still no clear understanding of the function of VDAC1 in the basal body, nor is it clear whether VDAC1 regulates mitochondrial VDAC. Fatty acid-binding proteins 4 (FABP4) is an intracellular lipid chaperone that can reversibly bind hydrophobic ligands, and play an important role on carrying fatty acids to several organelles [31]. It is well established that circulating serum FABP4 is positively associated with body mass index and exerts hormonal regulations in obesity-associated diseases [32–34]. In addition, FABP4 plays a malignant role in metastatic cancers [35, 36], especially in THCA [37]. It was consistent with our conclusion in this study. Isocitrate dehydrogenase 2 (IDH2) is a member of the IDH family which is a key component of the tricarboxylic acid cycle [38, 39]. It was always reported as oncogene and played an important role in the biological mechanisms and progression in multiple tumor types [40]. IDH2 mutant inhibitors will probably improve the clinical treatment of certain cancer type [41–43]. Thyroglobulin (*T*g) is a macromolecular glycoprotein secreted by thyroid follicular epithelial cells, most of which are synthesized by thyroid cells and released into the residual cavity of thyroid follicles [44, 45]. It can be used as an important reference index for follow-up of patients with differentiated thyroid cancer after treatment. These results suggest that the five-protein nomogram may be a potential biomarker for predicting prognosis. However, the detailed predictive performance needs to be further validated in multicenter studies and larger cohorts in the future.

In conclusion, this study identified potential molecular mechanisms underlying the progression of PTMC. Machine learning suggested potential diagnostic biomarkers for PDLIM4, ANXA1, PKM, NPC2, LMNA, and FN1. Finally, the five-protein nomogram may be a prognostic biomarker in the future. However, the predictive ability and biological function need to be further verified by experiments with larger sample size and clinical trials. We hope that our study will provide a theoretical basis for clinical development and progress in the diagnosis and evaluation of PTMC.

## Supplementary Information

Below is the link to the electronic supplementary material.

Supplementary file1 (PDF 85 KB)

## Declarations

### Data availability

Relative data are available on request from the corresponding authors.
