# Supplementary material for: Identification of potential diagnostic and prognostic biomarkers for papillary thyroid microcarcinoma (PTMC) based on TMT-labeled LC–MS/MS and machine learning
Source: J Endocrinol Invest. 2022 Nov 23;46(6):1131–43. doi: 10.1007/s40618-022-01960-x (PMC10185636; doi:10.1007/s40618-022-01960-x)
Supplement: Supplementary file 1 — Supplementary file1 (PDF 85 KB) [file 40618_2022_1960_MOESM1_ESM.pdf]

Table 1 Clinical and histopathological features of PRM patients

Case	Age	Gender	Tumor size(mm)	Stage at diagnosis
1	33	M	10	N1a
2	49	F	10	N1a
3	27	M	6	N1b
4	24	M	5	N1b
5	27	M	10	N1a
6	34	F	8	N1a
7	33	F	10	N1a
8	33	M	6	N1a
9	32	F	7	N1a
10	34	M	10	N1a
11	44	F	10	N0
12	52	F	6	N0
13	53	F	10	N0
14	48	F	9	N0
15	41	F	8	N0
16	43	F	10	N0
17	44	F	8	N0
18	55	F	10	N0
19	57	F	10	N0
20	34	F	3	N0
